# The Efficacy of Acupuncture in Post-Operative Pain Management: A Systematic Review and Meta-Analysis

**DOI:** 10.1371/journal.pone.0150367

**Published:** 2016-03-09

**Authors:** Ming-Shun Wu, Kee-Hsin Chen, I-Fan Chen, Shihping Kevin Huang, Pei-Chuan Tzeng, Mei-Ling Yeh, Fei-Peng Lee, Jaung-Geng Lin, Chiehfeng Chen

**Affiliations:** 1 Division of Gastroenterology, Department of Internal Medicine, Wan Fang Hospital, Taipei Medical University, Taipei, Taiwan; 2 Division of Gastroenterology and Hepatology, Department of Internal Medicine, School of Medicine, College of Medicine, Taipei Medical University, Taipei, Taiwan; 3 Department of Nursing, Wan Fang Hospital, Taipei Medical University, Taipei, Taiwan; 4 School of Nursing, College of Nursing, Taipei Medical University, Taipei, Taiwan; 5 Cochrane Taiwan, Taipei Medical University, Taipei, Taiwan; 6 Institute of Management of Technology, National Chiao Tung University, Hsinchu, Taiwan; 7 Evidence-Based Medicine Center, Wan Fang Hospital, Taipei Medical University, Taipei, Taiwan; 8 Graduate Institute of Integration of Traditional Chinese Medicine with Western Nursing, National Taipei University of Nursing and Health Sciences, Taipei, Taiwan; 9 Graduate Institute of Clinical Medicine, College of Medicine, Taipei Medical University, Taipei, Taiwan; 10 Department of Otolaryngology, Taipei Medical University-Shuang Ho Hospital, New Taipei City, Taiwan; 11 School of Chinese Medicine-Acupuncture Science, China Medical University, Taichung, Taiwan; 12 Department of Public Health, School of Medicine, College of Medicine, Taipei Medical University, Taipei, Taiwan; 13 Division of Plastic Surgery, Department of Surgery, Wan Fang Hospital, Taipei Medical University, Taipei, Taiwan; University of Bari, ITALY

## Abstract

**Background:**

Postoperative pain resulting from surgical trauma is a significant challenge for healthcare providers. Opioid analgesics are commonly used to treat postoperative pain; however, these drugs are associated with a number of undesirable side effects.

**Objective:**

This systematic review and meta-analysis evaluated the effectiveness of acupuncture and acupuncture-related techniques in treating postoperative pain.

**Data Source:**

MEDLINE, Cochrane Library, and EMBASE databases were searched until Sep 30, 2014.

**Study Eligibility Criteria:**

Randomized controlled trials of adult subjects (≥ 18 years) who had undergone surgery and who had received acupuncture, electroacupuncture, or acupoint electrical stimulation for managing acute post-operative pain were included.

**Results:**

We found that patients treated with acupuncture or related techniques had less pain and used less opioid analgesics on Day 1 after surgery compared with those treated with control (P < 0.001). Sensitivity analysis using the leave-one-out approach indicated the findings are reliable and are not dependent on any one study. In addition, no publication bias was detected. Subgroup analysis indicated that conventional acupuncture and transcutaneous electric acupoint stimulation (TEAS) were associated with less postoperative pain one day following surgery than control treatment, while electroacupuncture was similar to control (P = 0.116). TEAS was associated with significantly greater reduction in opioid analgesic use on Day 1 post surgery than control (P < 0.001); however conventional acupuncture and electroacupuncture showed no benefit in reducing opioid analgesic use compared with control (P ≥ 0.142).

**Conclusion:**

Our findings indicate that certain modes of acupuncture improved postoperative pain on the first day after surgery and reduced opioid use. Our findings support the use of acupuncture as adjuvant therapy in treating postoperative pain.

## Introduction

Postoperative pain results from surgical trauma and is a significant challenge for healthcare providers [1]. About 75% of patients experience moderate or severe pain following surgery [2]. The mainstay of treating postoperative pain is the use of opioid analgesics such a morphine, hydromorphine, meperidine, or fentanyl [3]. However, these drugs are associated with a number of undesirable side effects which can delay patient recovery including nausea, vomiting, dizziness, sedation, and decreased gut motility [3,4]. The use of customized strategies for administering analgesic, for example patient controlled analgesia, is designed to reduce consumption of opioid analgesics and have resulted in better pain control [5]. However, even with individualized pharmacological approaches for treating postoperative pain, the side effects of opioid analgesics remain high [2].

Acupuncture is often used to treat pain, and numerous studies have found it is safe compared to routine care [6–9]. A number of clinical studies have evaluated the efficacy of acupuncture and related methods as adjuvant treatment for postoperative analgesia [1]. Two prior meta-analyses evaluated the use of acupuncture in treating postoperative pain [1,8]. One focused on the use of acupuncture following back surgery [8]. The other, which was performed in 2008, evaluated the use of acupuncture more broadly following surgery [1]. Since the publication of these two meta-analyses, additional trials have evaluated the use of acupuncture as adjuvant therapy in treating postoperative pain. In this systematic review and meta-analysis, we further evaluated the effectiveness of acupuncture and acupuncture-related techniques in treating postoperative pain.

## Materials and Methods

### Search strategy

This meta-analysis was conducted in accordance with PRISMA guidelines [10]. MEDLINE, Cochrane Library, and EMBASE databases were searched until Sep 30, 2014. The following search terms were used: acupuncture, electroacupuncture, acupoint electrical stimulation, postoperative pain. Randomized placebo or sham controlled trials of adult subjects (≥ 18 years) who had undergone surgery and received acupuncture, electroacupuncture, or acupoint electrical stimulation for managing acute post-operative pain were included. Studies that evaluated auricular acupuncture were excluded. Only papers published in English or Chinese were included. Letters, comments, editorials, care reports, technical reports, or any non-original studies were excluded. Studies were also excluded if the outcomes of interest were not presented quantitatively.

The review and selection of studies, study data extraction, and quality assessment were hand-searched by two independent reviewers, and if necessary a third reviewer was consulted to resolve any uncertainties regarding the inclusion.

### Data extraction and quality assessment

The following information was extracted from studies that met the inclusion criteria: the name of the first author, year of publication, study design, demographic data of subjects, types of intervention, and outcomes. The quality of the data was evaluated using the Cochrane Risk of Bias Tool [11].

### Statistical analysis

The primary outcome was the pain score on the first day (Day 1) following surgery. The secondary outcome was the cumulative use of opioid analgesics in the acupuncture and control treatment groups. The cumulative use of opioid analgesics was defined as the cumulative amount (sum) within 24 hours after surgery (mg) In addition, planned subgroup analysis of treatment effectiveness was performed according to difference interventions (ie, acupuncture eletroacupuncture, transcutaneous electric acupoint stimulation (TEAS), and control).

For the different outcomes, mean with standard deviations (SDs) were calculated and were compared between treatment groups. If the median and interquartile range (IQR) was reported in a study, we assumed that the median of the outcome variable was equal to the mean response and width of the interquartile range was approximately 1.35 standard deviation [11]. Difference in means with 95% confidence intervals (CIs) for each individual and combined study was calculated for pain score at Day 1 following surgery. Because various opioid analgesics were used, standardized difference in means (SDM) with corresponding 95% CIs were calculated for each individual study and for the studies combined. A χ2-based test of homogeneity was performed and the inconsistency index (I^2^) and Q statistics were determined. If the I^2^ statistic was > 50%, a random-effects model was used. Otherwise, a fixed-effect model was employed. Pooled effects were calculated and a 2-sided P value < 0.05 was considered to indicate statistical significance.

Sensitivity analysis was carried out for primary outcome using the leave-one-out approach. Publication bias was also assessed by determining if the data points formed a symmetric funnel-shaped distribution and had a one-tailed significance level P > 0.05 (Egger’s test). All analyses were performed using Comprehensive Meta-Analysis statistical software, version 2.0 (Biostat, Englewood, NJ, USA).

## Results

### Literature Search

**Fig 1:**
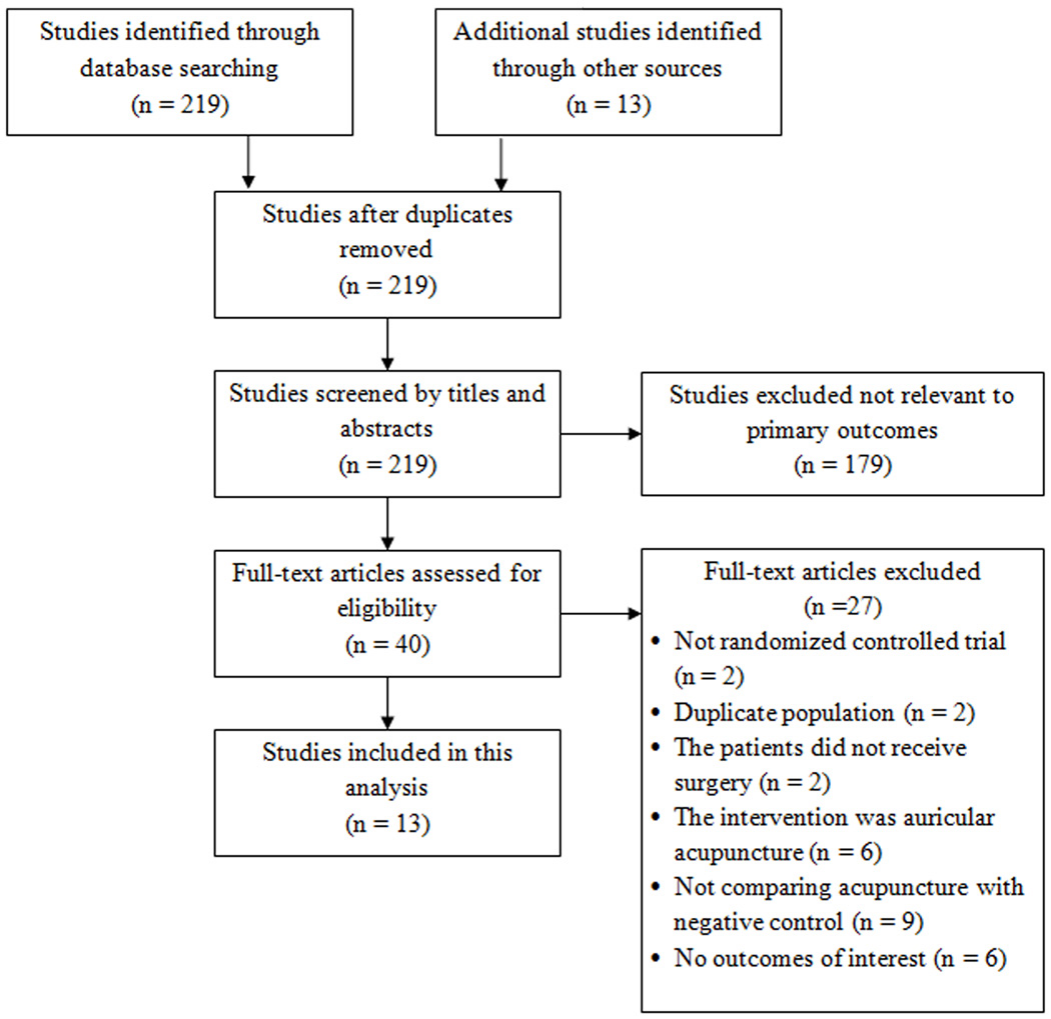
Flowchart of study selection.

Of the 219 articles identified in the initial search, 179 were excluded for not being relevant (Fig 1). Forty were fully reviewed and 27 of these were excluded due to not being randomized controlled trials (n = 2), reporting findings from the same patient population (n = 2), patients did not receive surgery (n = 2), the intervention was auricular acupuncture (n = 6), not having a control arm (n = 9), or not reporting outcomes of interest (n = 6). Thirteen studies were included for qualitative and quantitative analyses [12–24].

### Study characteristics

The 13 studies included 682 patients; 384 patients received acupuncture or related treatments and 298 were treated with control (Table 1). The mean age of patients in the acupuncture group ranged from 40–76 years and in the control group ranged from 41–75 years. Four studies included only female patients [12,18–20]. The location of the acupuncture varied across studies as did the timing of when the treatment was given relative to surgery; eight studies treated patients with acupuncture after surgery, three treated patients before surgery, and two treated patients both before and after surgery. In most (9/11) of the studies providing pain score data, VAS that assessed pain was used. The two studies that did not use VAS were Langenbach et al. (2012) [17] who used the Numeric Rating Scale (NRS)-11 [25] and Kotani N et al. (2001) [15] who used percentage in pain relief which conceptually is similar to the linear spectrum of VAS.

**Table 1:** Summary of basic characteristics of the included studies.

First author (year)	Type of surgery	Sample size	Time point	Frequency	Acupoints	Age (yrs)	Female (%)
***Acupuncture vs*. *control***							
Ward (2013)	Arthroscopic shoulder surgery	10/12	after surgery	Once, on op day	GB 21(Jinajing), LU 1 (Zhongfu), distal LI 11 (Quchi), LI 4 (Heu), TE 3 (Zhongzhu), TE 5 (Waiuan)	51 vs. 46	70% vs. 58%
Langenbach (2012)	Hemorrhoidopexy	17/16	after surgery	afternoon on op day, every morning and afternoon post-op day 1, day 2	Du2 (yao shu), Du20 (bai hui), Bi30 (bai han shu), Bi57 (cheng shan), Ma44 (nei ting), Pe6 (nei guan)	62 vs. 48	53% vs. 47%
Kotani (2001)	Abdominal surgery	89/86	before surgery	On the day before surgery	T9–L3 spinal vertebrae (BL18–BL24) for upper abdominal surgery; the T11–L5 spinal vertebrae(BL20–BL26) for lower abdominal surgery	53 vs. 55	37% vs. 36%
Wang (2000)	Lumbar disc protrusion surgery	132	before and after surgery	2–3 times for a total of 3–6 days	De-Qi sensation	21–28	41%
***Eletroacupuncture vs*. *control***							
Coura (2011)	Cardiac surgery	13/9	before surgery	once	bilateral LI4–LI11, LR3–ST36, PC6–TE5	56 vs. 63	23% vs. 56%
Wong (2006)	Thoracotomy	13/12	after surgery	twice daily for the first 7 post-op days	LI 4, GB 34, GB 36, and TE 8	65 vs. 65	37% vs.17%
Lin (2002)	Lower abdominal surgery	50/25	after surgery	once	Zusanli acupoints (ST36)	40 vs. 41	100% vs. 100%
Sim (2002)	Gynaecologic lower abdominal surgery	60/30	before and after surgery	once	ST36 and PC6	46 vs.47	100% vs. 100%
***TEAS vs*. *control***							
Lan (2012)	Total hip arthroplasty	30/30	before surgery	before incision, and at post-op 2h, 4h, 20h, 44h	bilateral P6, L14 and ipsilateral to the surgery site ST36, GB31	76 vs. 75	47% vs. 43%
Yeh (2011)	Spinal surgery	30/30	after surgery	post-op 2h, 3h	Weizhong (BL40), Yanglingquan (GB34), Shenmen (HT7), and Neiguan (P6)	61 vs. 57	67% vs. 70%
Chiu (1999)	Hernorrhoidectomy	30/30	after surgery	two times per day	Hegu, Lieque	53 vs. 56	33% vs.17%
Chen (1998)	Major gynecologic surgery	25/25	after surgery	once	Zusanli acupoints (ST36)	43 vs. 45	100% vs. 100%
Wang (1997)	Lower abdominal surgery	50/25	after surgery	once	Hegu (LI 4), a second set of electrodes was placed on either side of the surgical incision	44 vs. 44	100% vs. 100%

Abbreviation: op = operation, TEAS: transcutaneous electrical stimulation.

### Quality assessment, sensitivity analysis, and publication bias

**Fig 2:**
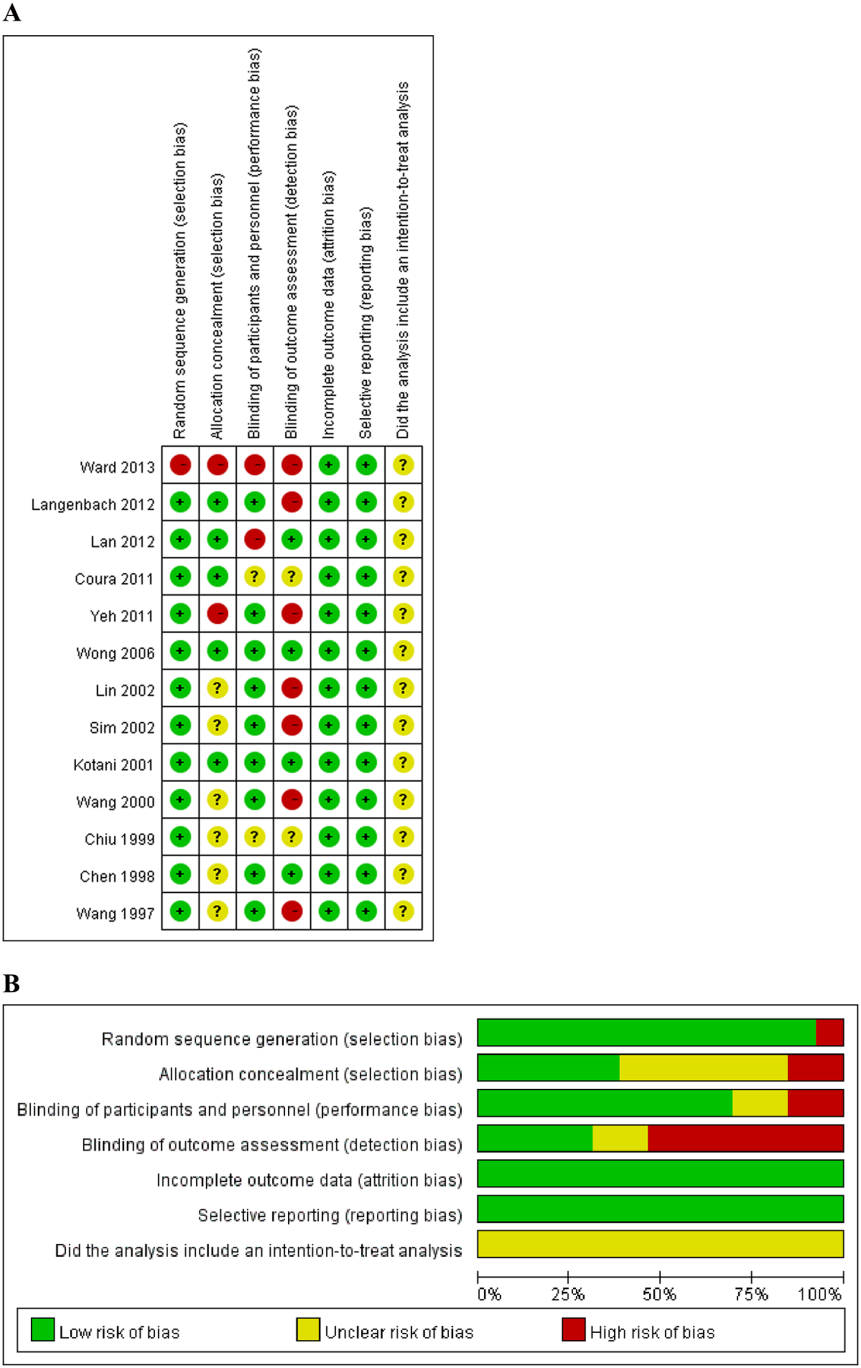
Results of quality assessment of included randomized controlled trials or prospective comparative studies. (A) Potential risk of bias of each included study. (B) Summarized risk of included studies.

The quality of the data was moderate. In over half the studies there were a high risk of detection bias due to the studies and the allocation of treatment not being adequately blinded (Fig 2A and 2B). In all of the studies, it was unclear if an intention-to-treat analysis was performed.

**Fig 3:**
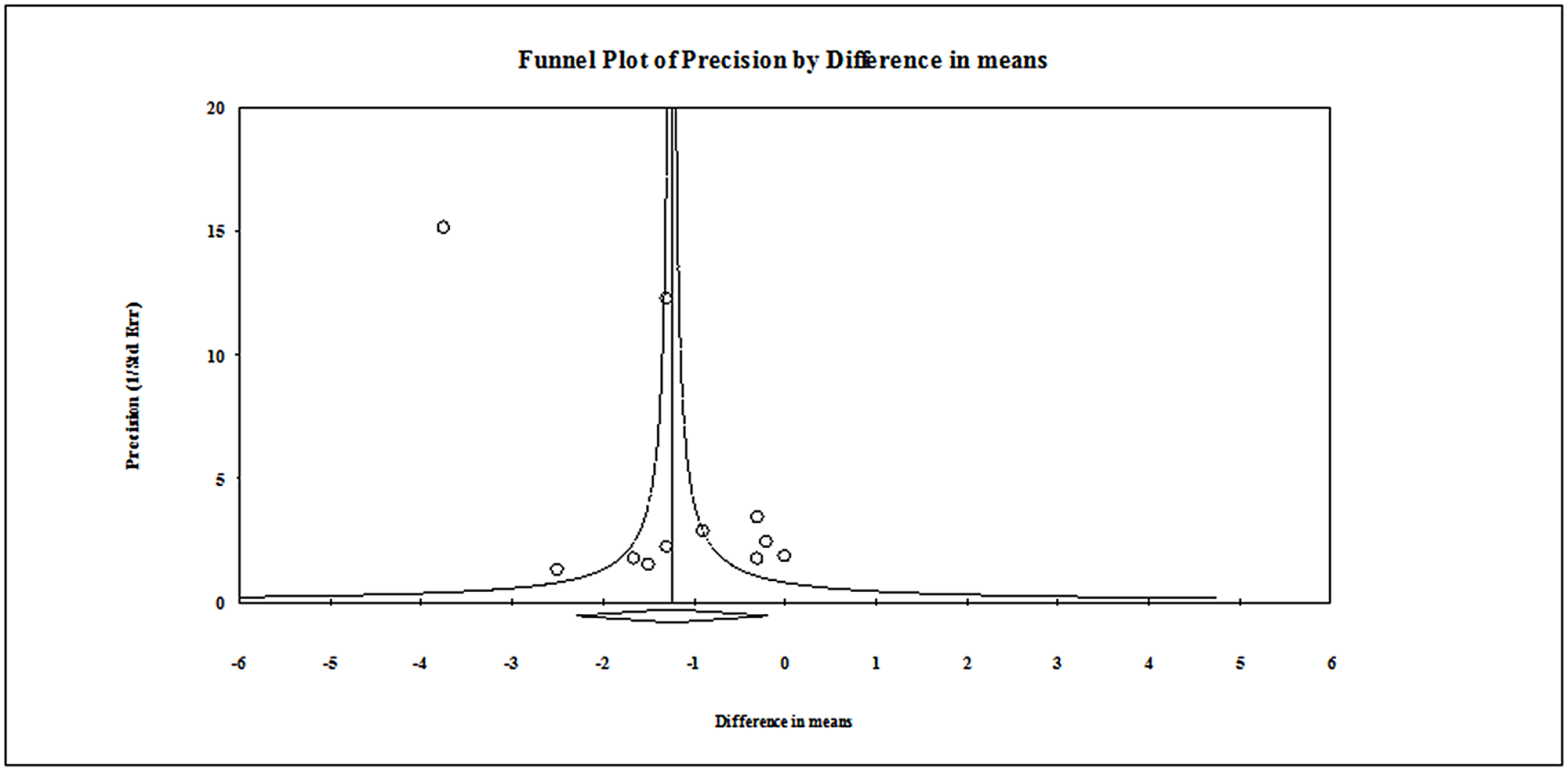
Publication bias for pain score at Day 1 post-surgery.

Sensitivity analysis was done using the leave-one-out approach. The direction of the combined estimates did not vary markedly with the removal of each study in turn, indicating that the meta-analysis was robust and the data was not overly influenced by any one study (Table 2). The results via Egger’s test showed there was no publication bias for the findings with regard to pain score on Day 1 following surgery (t = 1.588, one-tailed, P = 0.073, Fig 3)

**Table 2:** Sensitivity analysis for pain score on Day 1 post-surgery.

Study name	Statistics with study removed				
	Difference in means	Lower limit	Upper limit	Z-Value	P-Value
***Acupuncture vs*. *control***					
Langenbach (2012)	-1.24	-2.35	-0.13	-2.19	0.028
Wang (2000)	-0.92	-1.34	-0.50	-4.30	0.000
***Eletroacupuncture vs*. *control***					
Coura (2011)	-1.22	-2.33	-0.12	-2.18	0.029
Wong (2006)	-1.37	-2.46	-0.28	-2.46	0.014
Lin (2002)	-1.21	-2.31	-0.10	-2.14	0.032
Sim (2002)	-1.35	-2.45	-0.26	-2.43	0.015
***TEAS vs*. *control***					
Lan (2012)	-1.35	-2.44	-0.26	-2.42	0.016
Yeh (2011)	-1.28	-2.39	-0.17	-2.27	0.023
Chiu (1999)	-1.24	-2.57	0.08	-1.84	0.066
Chen (1998)	-1.14	-2.24	-0.04	-2.03	0.043
Wang (1997)	-1.34	-2.44	-0.24	-2.39	0.017

Abbreviation: TEAS: transcutaneous electrical stimulation.

### Meta-analysis

**Fig 4:**
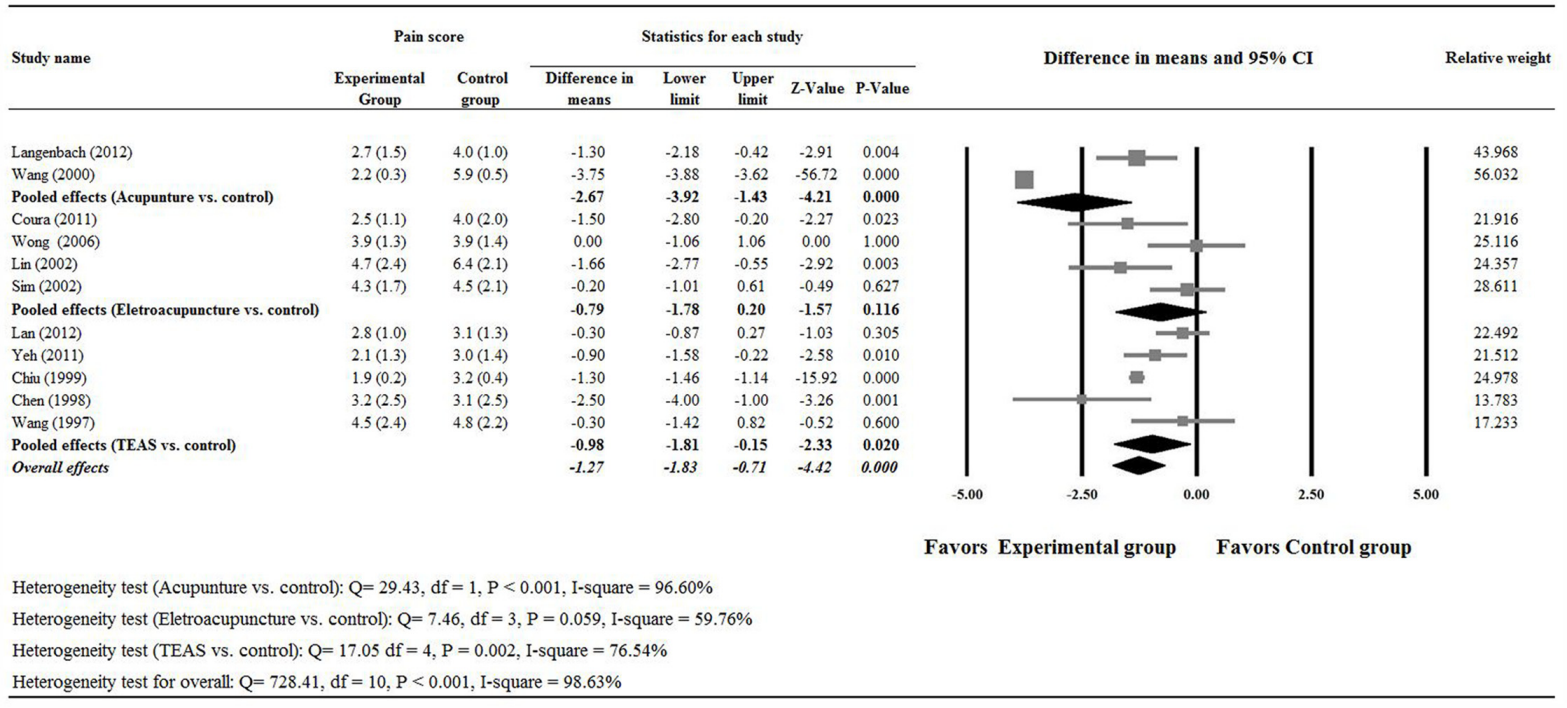
Meta-analysis for pain score on the first day after surgery.

Eleven [12–14,16–21,23,24] of the 13 studies provided completed numerical data for pain score on the first day post-surgery (Day 1) between experimental and control groups and were included in the meta-analysis. There was evidence of heterogeneity among the 11 studies (Q statistic = 728.418, I^2^ = 98.63, P < 0.001); therefore, a random-effects model of analysis was used. The pooled difference in means (-1.27, 95% CI = -1.83, -0.71) indicated that patients in the experimental group had less pain on Day 1 than those in the control group (P < 0.001, Fig 4).

**Fig 5:**
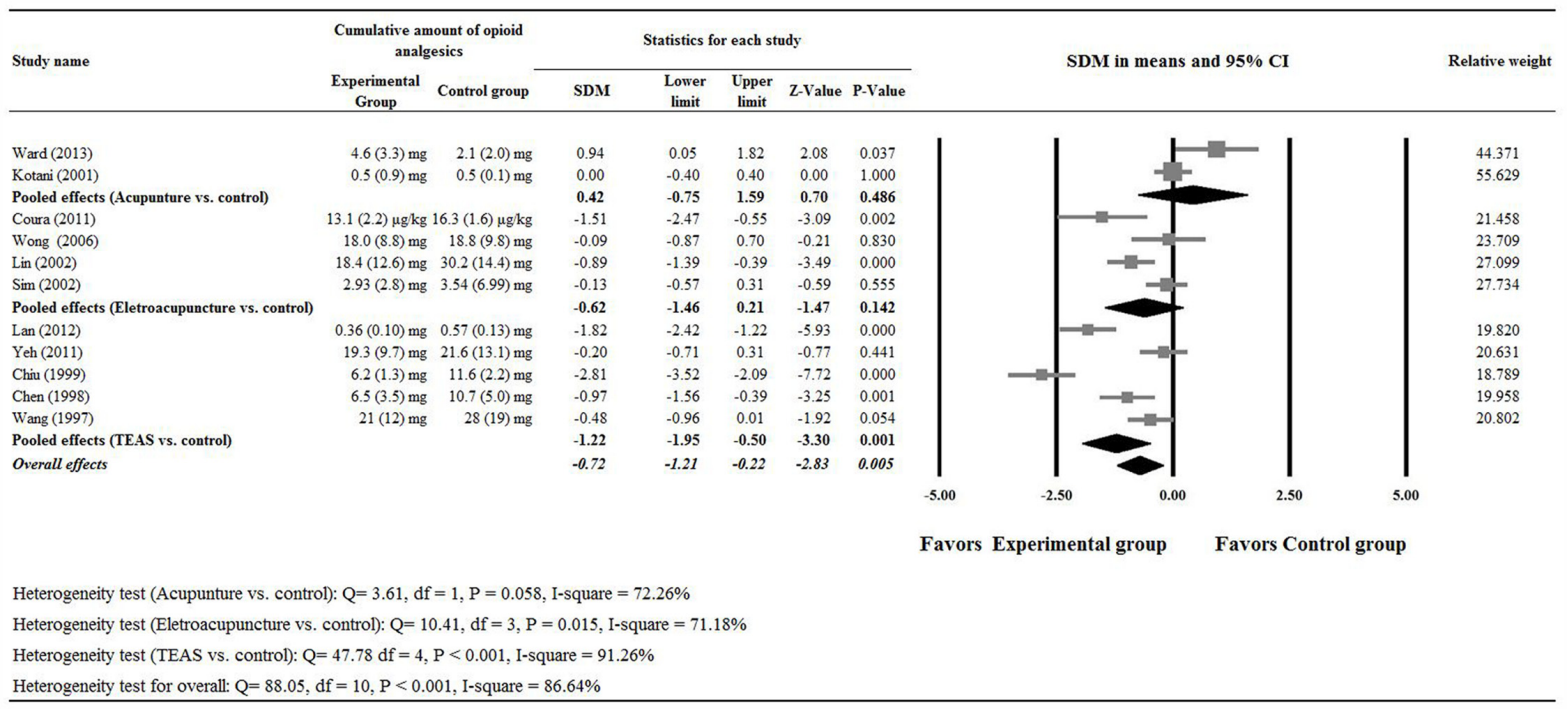
Meta-analysis for the cumulative amount of opioid analgesics used.

Eleven of the studies [12–16,18–20,22–24] provided completed numerical data for the cumulative amount of opioid analgesics and were included in the meta-analysis. Analysis of the data indicated there was heterogeneity (Q = 88.05, P < 0.001 and I^2^ = 86.64%); hence, a random effect model was used. The meta-analysis of the pooled data showed patients in the experimental group had less opioid analgesics usage than those in the control group (SDM, -0.72; 95% CI = -1.21, -0.22, P = 0.005) (Fig 5).

### Subgroup Analysis

#### Acupuncture vs. control

Two studies compared acupuncture and control treatments [17,21] were included in the subgroup analysis that evaluated these two treatments. A random- effects model was used for the analysis since heterogeneity in the data was detected (heterogeneity: Q = 29.43, P < 0.001 and I^2^ = 96.60%). The meta-analysis found patients in the experimental acupuncture group had less pain than those in the control group (difference in means, -2.67; 95% CI of -3.92, -1.43, P < 0.001) (Fig 4). However, both groups used similar amounts of opioid analgesics (SDM: 0.42; 95% CI of -0.75, 1.59, P = 0.486, Fig 5).

#### Electroacupuncture vs. control

Four studies evaluated electroacupuncture with control therapy [14,18,19,23] and were included in the subgroup analysis that assessed the relative efficacy of these two therapies. Random- effects models were used for the analysis on pain score and cumulative amount of opioid analgesics used since heterogeneity in the data were detected (For pain score, heterogeneity: Q = 7.46, P = 0.059 and I^2^ = 59.76%; and for cumulative amount of opioid analgesics used, heterogeneity: Q = 10.41, P = 0.015 and I^2^ = 71.18%). The pooled meta-analysis of data showed there were no significant differences in the pain score (difference in means, -0.79; 95% CI of -1.78, 0.20, P = 0.116, Fig 4) and cumulative amount of opioid analgesics used (SDM: -0.62; 95% CI of -1.46, 1.21, P = 0.142, Fig 5) on Day 1 post surgery between the two treatment groups.

#### Transcutaneous electric acupoint stimulation (TEAS) vs. control

Five studies [12,13,16,20,24] reported outcomes of TEAS compared with control were used in subgroup analysis that compared these two treatments. There was evidence of heterogeneity among the five studies (Q statistic = 17.05, I^2^ = 76.54, P = 0.002); therefore, a random-effects model of analysis was used. The pooled difference in means (-0.98, 95% CI = -1.81, -0.15) indicated that patients who were treated with TEAS had less pain on the first day after surgery than those treated with control (P = 0.020, Fig 4). In addition, the pooled meta-analysis showed patients in the TEAS group had less opioid analgesics use than those in the control group (SDM: -1.22; 95% CI of -1.95, -0.50, P = 0.001, heterogeneity: Q = 47.78, P < 0.001 and I^2^ = 91.26%, Fig 5).

## Discussion

This systematic review and meta-analysis assessed the effectiveness of acupuncture and acupuncture-related methods in treating postoperative pain. We found that patients treated with acupuncture, or related techniques, had less pain and used less opioid analgesics on the first day after surgery compared with those treated with control (P < 0.001). Sensitivity analysis using the leave-one-out approach indicated the findings are robust and not dependent on any one study. In addition, no publication bias was detected. Subgroup analyses suggest that conventional acupuncture and TEAS were associated with less postoperative pain than control treatment on Day 1 post surgery, while electroacupuncture was similar to control (P = 0.116). TEAS was associated with significantly greater reduction in opioid analgesic use on first day after surgery than control (P < 0.001); however, conventional acupuncture and electroacupuncture showed no benefit in reducing opioid analgesic use compared with control (P ≥ 0.142). Our findings indicate that certain methods of acupuncture improved postoperative pain a day after surgery, supporting the use of acupuncture as adjuvant therapy in treating postoperative pain.

A number of studies have shown that acupuncture or electrical stimulation in specific frequencies applied to certain body sites can facilitate the release of specific neuropeptides in the CNS, eliciting profound physiological effects and even activating self-healing mechanism [26]. Acupuncture analgesia affects the peripheral and central pain pathways [27]. Apart from the mechanisms upon pain threshold elevation and pain pathway inhibition, various endogenous neuropeptides act in central processing in the brain to create analgesia. Opioids, including β-endorphins, enkephalins, and dynorphins release have been shown to cause analgesia by acting in the CNS. Cholecystokinin (CCK) octapeptide, 5-hydroxytryptamine (5-HT) and N-methl-D-aspartic acid (NMDA) receptor inhibition have strong associations with the analgesic effect [26,28]. Several other neurotransmitters are being investigated for acting in the analgesic pathway, such as noradrenalin, γ-amino-butyric acid (GABA), and substance P.

Only one previous meta-analysis evaluated the efficacy of acupuncture and related techniques as adjunctive therapy for acute postoperative pain management [1]. Sun et al. (2008) included 15 studies, which compared acupuncture with control, and included 1166 patients of whom 608 received acupuncture. The types of acupoint stimulation assessed were acupuncture, moxibustion, TEAS, and acupressure. Consistent with our results, they found postoperative pain intensity, as measured by a visual analogue scale (0–100 mm), was significantly reduced in the acupuncture group compared with control group at eight hours after surgery (weighted mean difference [95% CI]; -14.57 mm [-23.02, -6.13] and 72 hours (-9.75 mm [-13.82, -5.68]), but not at 24 hours post-surgery (-5.59 mm [-11.97, 0.78]). Also, similar to our findings, they found that acupuncture was associated with a greater reduction in cumulative use of opioid analgesics for postoperative pain at eight hours (weighted mean difference [95% CI]; -3.14 mg [-5.15, -1.14], 24 hours (-8.33 mg [-11.06, -5.61]), and 72 hours (-9.14 mg [-16.07, -2.22) following surgery compared with control. Across the studies included in the Sun et al. meta-analysis, the relative reduction in opioid use with acupoint stimulation was 21%-29% which is generally considered clinically significant [1]. Of note, both for pain and opioid use, different studies were pooled for each timepoint (ie, 8, 24, and 72 hours) which could have affected the findings [1]. The study of Sun et al. included 15 studies while our study only included 11. The difference reflects that we did not include studies which evaluated auricular acupuncture because the methodology and principles in mechanism of action differ from the traditional Chinese medicine.

The acupuncture group in Sun et al was associated with a lower incidence of opioid-related side effects including nausea, dizziness, sedation, pruritus, and urinary retention. The reduction in opioid-related adverse events supports the clinical importance of the acupuncture treatment. Moreover, side effects attributed to acupuncture were mild and resolved spontaneously. We did not assess opioid-related side effects in our analysis.

The mechanism of acupuncture analgesia is not clear. Acupuncture may depress pain by activating a number of neurotransmitters or modulators such as opioid peptides, norephinephrine, serotonin, and adenosine [29]. Acupuncture may also activate the endogenous pain inhibitory pathway [1,29].

There are several limitations to our analysis. The types of acupoints and surgical procedures used in the included studies were heterogeneous which may have affected the findings. The studies used different control models, which may influence the results. Currently, it is unclear what control model(s) might be the best. Some studies may even have more than 1 control models within one study for better comparison. 2 studies used standard care without any acupuncture device as the control group. Some studies had sham control as acupunctures or electroacupunctures were applied at non-acupuncture points. There were also studies using needles attached to the skin without skin penetration. Other studies used sham electroacupuncture with lower electric frequency or without current flow at all. In addition, the studies differed in design and in several of the studies there was inadequate blinding of patients and outcome assessment. We only analyzed findings from the first day following surgery and additional time points are necessary to better understand the benefit of acupuncture and related methods for managing postoperative pain. We also did not evaluate if acupuncture reduced the side effects of opioid analgesics. Three types of pain measurements were used across the studies: VAS (9 of the studies) and NRS-11 and percentage in pain relief (1 study each). The use of different utilities for measuring pain might impact some of the evaluation and degree of pain assessments. We also did not adjust the treatment variation between studies. Future studies are needed to further explore these findings.

Our study found that acupuncture and related techniques one day following surgery significantly reduced postoperative pain and the use of opioids. Unlike Sun et al, we also performed subgroup analysis and found that conventional acupuncture and TEAS may be more efficacious than electroacupuncture as adjuvant therapy to manage postoperative pain. Additional well designed studies are required to further explore the role of acupuncture and related methods in treating postoperative pain.

## Supporting Information

S1 PRISMA Checklist PRISMA Checklist. (DOC)
